# Synergistic effects of triglyceride-glucose index and body mass index combined with depression in predicting stroke events: a study based on two national cohorts

**DOI:** 10.3389/fnut.2025.1633655

**Published:** 2025-09-03

**Authors:** He Jian, Luo Jie, YuanDing Jiang, YongHong Duan, Wang Bing, RiChu Liang, ZhenKun Xiao, JiaHui Zhang, Tang Ting

**Affiliations:** ^1^Department of Neuromedicine Center, The Second Affiliated Hospital, University of South China, Hengyang, Hunan, China; ^2^Hengyang Medical School, University of South China, Hengyang, Hunan, China; ^3^Health Management Center, The Second Affiliated Hospital, University of South China, Hengyang, Hunan, China; ^4^Department of Teaching and Student Affairs, The Second Affiliated Hospital, University of South China, Hengyang, Hunan, China

**Keywords:** triglyceride-glucose index, body mass index, TyG-BMI, stroke, depression

## Abstract

**Background:**

Stroke risk associated with the triglyceride-glucose index-body mass index (TyG-BMI) has been increasingly recognized. Depression has also been firmly established as a factor related to the development of stroke. However, there remains a research gap in evaluating the combined effect of TyG-BMI and depression on the risk of stroke. This study aims to address the inconsistency between TyG-BMI, depression, and stroke incidence.

**Methods:**

This study utilized longitudinal data from the China Health and Retirement Longitudinal Study (CHARLS), involving 6,417 participants, and the National Health and Nutrition Examination Survey (NHANES) database, which included data from 17,754 participants. The analytical approach involved applying Multivariate logistic regression analysis to assess the risk of stroke with the combined evaluation of TyG-BMI and depression. Additionally, we conducted smoothing curve fitting, subgroup analysis, interaction tests, and predictive modeling for further evaluation.

**Results:**

A total of 24,171 participants from two national cohorts were included in the analysis. Among them, 1,223 individuals had a history of stroke. Compared to individuals with lower TyG-BMI and no depression, those with higher TyG-BMI and depression exhibited a significantly higher risk of stroke. The restricted cubic spline (RCS) model indicated that the combination of elevated TyG-BMI and depression had a strong predictive value for stroke occurrence.

**Conclusion:**

The findings of this study suggest a positive interaction between TyG-BMI and depression in predicting stroke risk. The combined evaluation of TyG-BMI and depression should be emphasized to enhance primary prevention efforts for stroke.

## Introduction

Stroke is one of the leading causes of death globally (1). It is also one of the most fatal diseases in both the United States and China, with its incidence showing a marked increasing trend since 1990 (2). The adverse outcomes of stroke impose a significant economic burden on families and society (3, 4).

Recently, insulin resistance (IR), characterized by reduced sensitivity or reactivity to insulin’s metabolic effects, including insulin-mediated glucose handling, has been widely recognized as an independent risk factor for stroke (5, 6). The metabolic and lipid disturbances caused by IR lead to long-term damage to the vascular wall, triggering atherosclerosis, plaque formation, and subsequent narrowing of the vascular lumen and decreased vascular wall elasticity, ultimately resulting in the onset of stroke (7–9).

The triglyceride-glucose (TyG) index has gained recognition in numerous studies as a reliable surrogate marker for insulin resistance (IR) (10, 11), a major risk factor for cardiovascular and cerebrovascular diseases. Recent research has highlighted the predictive value of the TyG index for stroke risk, underscoring its significant role in identifying individuals at higher risk for cerebrovascular events (12, 13). The TyG index is also strongly correlated with well-established stroke risk factors, including atherosclerosis and hypertension, further reinforcing its relevance in stroke risk prediction (5).

The global prevalence of high body mass index (BMI) has reached alarming levels and is strongly associated with various metabolic disorders such as insulin resistance, glucose intolerance, and metabolic syndrome (14, 15). These conditions significantly contribute to the initiation and progression of cerebrovascular diseases, ultimately impacting prognosis and outcomes for stroke patients (16, 17). When combined, TyG and BMI have been shown to interact in a way that exacerbates metabolic dysfunction, with previous studies demonstrating that this combined effect is linked to heightened stroke risk (18, 19).

In parallel, depression has emerged as a crucial independent factor for stroke risk. The American Heart Association has emphasized that individuals with depression are at a significantly higher risk for coronary artery disease, cerebrovascular disease, and stroke (20, 21). A growing body of evidence, including meta-analyses by Meng et al. (22), has provided compelling insights into the strong association between depression and cardiovascular disease, further solidifying its role as an important risk factor for stroke (23, 24).

While the individual associations between TyG-BMI, depression, and stroke risk are well established, no study has yet explored their combined effects on stroke risk. Given that both metabolic dysfunction and depression are prevalent and often co-exist in at-risk populations, we believe it is critical to investigate their synergistic impact on stroke risk. The absence of research on this intersection presents a significant gap in our understanding of stroke risk factors. By addressing this gap, we aim to provide valuable insights into how these two risk factors interact and contribute to stroke risk, which could ultimately inform more effective and comprehensive stroke prevention strategies.

Thus, we designed this study based on two large national cohorts to explore the combined relationship between TyG-BMI, depression, and stroke risk. We hope that this study will contribute to a deeper understanding of stroke risk factors and guide future public health interventions aimed at reducing stroke incidence.

## Methods

### Data sources and study population

This study utilizes data from two national cohort studies: the China Health and Retirement Longitudinal Study (CHARLS) and the National Health and Nutrition Examination Survey (NHANES).

CHARLS Cohort: CHARLS is a national, population-based cohort study initiated in 2011. It collects data biennially from Chinese adults across various regions. The study employs rigorous stratified sampling to recruit participants, and the data collected covers demographic characteristics, medical history, and lifestyle factors. Baseline data were gathered in 2011, with follow-up data collected through 2018. The study adheres to the principles of the Declaration of Helsinki and was approved by the Ethics Committee of Peking University (approval number: IRB00001052-11015) (25, 26).

NHANES Cohort: NHANES is a major health and nutrition survey conducted by the U.S. National Center for Health Statistics (NCHS). The NHANES study protocol was approved by the NCHS Institutional Review Board, and all participants provided informed consent. This cohort includes demographic, socioeconomic, health-related, and medical information, with data collected between 2011 and 2018. All information from NHANES is publicly available, and no additional ethical approval is required for its use.

**Figure 1:**
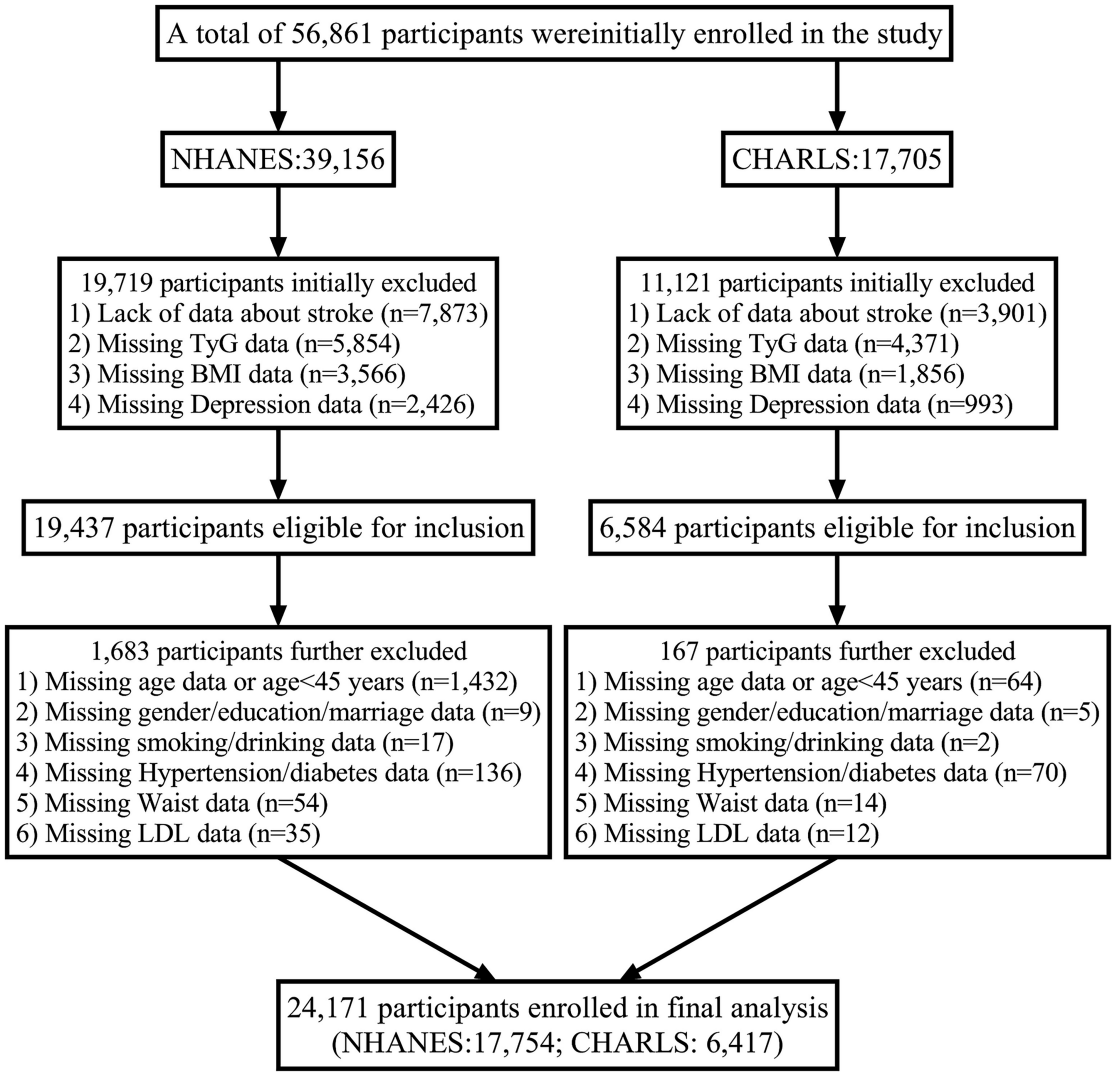
Flowchart of participant.

Both studies follow ethical research standards, and detailed inclusion and exclusion criteria for both cohorts are depicted in Figure 1.

### Assessment of depression

Depressive symptoms in CHARLS were assessed using the 10-item Center for Epidemiologic Studies Depression Scale (CESD-10). The scale consists of 10 questions, with a total score range from 0 to 30. A score of ≥12 is defined as “positive for depressive symptoms,” indicating clinically significant depression. In the NHANES database, the Patient Health Questionnaire-9 (PHQ-9) was used to assess depression. A score below 10 indicates no depression, while a score of 10 or higher indicates depression. The PHQ-9 is considered the most reliable screening tool for depression (27), with a sensitivity and specificity of 88% for detecting major depression at a cutoff of 10 (28).

### TyG-BMI calculation

The specific method for calculating TyG-BMI in this study is defined as follows: the TyG-BMI formula is TyGBMI = TyG × BMI, where TyG = ln [FPG (mg/dL) × TG (mg/dL) / 2], and BMI = weight / height^2^ (kg/m^2^) (18, 19).

### Assessment of stroke

The key diagnostic criterion for stroke in the CHARLS cohort is self-reported stroke, confirmed by a physician’s diagnosis, with the specific question posed to participants: “Has a doctor ever told you that you had a stroke?” In this cohort, participants with a prior history of stroke were excluded during the baseline survey in 2011 to ensure that only individuals without a history of stroke were included. All exposure variables (such as blood lipids and blood glucose) were measured at baseline (2011) or the first follow-up survey (2013). Stroke events strictly occurred after the measurement of exposures, ensuring the “exposure → outcome” temporal sequence. Follow-up intervals were 2 years, and new stroke events were anchored to specific months and years using the “date of first diagnosis.” Stroke events were monitored through follow-up surveys conducted in 2013, 2015, and 2018, where participants were again asked about their stroke history, and the date of their first stroke diagnosis was recorded (accurate to the month and year). If participants were lost to follow-up or deceased, information on stroke events was supplemented by responses from family members or relevant death certificates. Participants with a history of stroke at baseline, missing key variables, or lost to follow-up were excluded from the analysis.

On the other hand, the NHANES cohort adopts a cross-sectional approach for stroke diagnosis. Stroke was diagnosed through self-report with the question: “Has a doctor ever told you that you had a stroke?” Only participants reporting a stroke within the past year were included in the analysis, excluding those with a long-term stroke history to reduce recall bias. Additionally, stroke-related deaths between 2011 and 2018 were cross-validated with the National Death Index (NDI) using ICD-10 codes (I60–I69). Regarding the temporal sequence of exposure and outcome assessment, stroke status and biomarkers (e.g., blood lipids) were measured within the same survey cycle in the NHANES cohort. However, due to the lack of strict follow-up data in the NHANES cohort, weighted multivariable logistic regression was employed to calculate odds ratios (ORs) for this cohort in order to minimize biases between the two databases. The analysis was adjusted for geographic and population representation biases using complex sampling weights. Participants reporting a stroke occurring more than 1 year ago or missing key variables were excluded from the analysis.

### Covariates

The following covariates were included: (i) categorical variables: marriage, gender, home address, education, smoking, alcohol consumption, hypertension, use of antihypertensive medication, diabetes mellitus, use of hypoglycaemic medication; (ii) continuous variables: age, waist circumference, systolic blood pressure (SBP), diastolic blood pressure (DBP), fasting glucose (Glu), serum high-density lipoprotein cholesterol (HDL), serum low-density lipoprotein cholesterol (HDL), serum low-density lipoprotein (LDL), serum total cholesterol (TC), serum triglycerides (TG), and C-reactive protein (CRP).

### Data collection

The CHARLS project team employed a computer-assisted personal interview system for household surveys (29). The questionnaires covered demographic characteristics, health status, functional ability, diagnosed chronic diseases, and health-related behaviors. Interviewers also measured participants’ height, weight, and blood pressure, among other physiological indicators. Blood samples were collected by trained nurses, who obtained fasting venous blood samples from each participant. The samples underwent a complete blood count within 1–2 h of collection, followed by centrifugation to separate plasma from red blood cells. The samples were then aliquoted and stored at −20°C to ensure safe transportation. Ultimately, all blood samples were sent to the Chinese Center for Disease Control and Prevention for in-depth analysis (29).

The NHANES database used a multi-module design (questionnaires, physical exams, and laboratory tests) to collect data on gender, age, blood pressure, and blood samples. Standardized procedures were employed to ensure the quality of the data. Blood samples were processed, stored, and transported to the University of Minnesota in Minneapolis for analysis (30).

### Statistical analyses

Statistical analyses were performed using R software (version 4.4.3) and SPSS software (version 26.0), with statistical significance defined as a two-tailed *p*-value < 0.05. The baseline characteristics were categorized based on the median of TyG-BMI and the prevalence of depression, with group comparisons for baseline characteristics. Continuous variables were expressed as medians (interquartile range) or means (standard deviation, SD), while categorical variables were described by percentages and frequencies. Differences between TyG-BMI groups were assessed using the χ^2^ test, and differences in continuous variables were evaluated through analysis of variance and the Kruskal-Wallis H test.

To explore the relationships between TyG-BMI, depression, and stroke risk, multivariate logistic regression analyses were conducted. Three models were constructed: Model 1: unadjusted; Model 2: adjusted for age, sex, marriage, education level, smoking status, drinking, hypertension and diabetes; Model 3: further adjusted for Waist, SBP, DBP, Glu, HDL, LDL, TC, TG and CRP.

In order to address potential heterogeneity between the CHARLS and NHANES cohorts, meta-regression analysis was incorporated to assess and adjust for differences across the two cohorts, thereby enhancing the robustness of the results.

Furthermore, to examine the interaction between TyG-BMI and depression in relation to stroke risk, the study employed additional models to calculate the Excess Relative Hazard Ratio (RERI), the interaction attribution ratio (AP), and the interaction index (S), which allow for a more accurate representation of the synergistic effects between TyG-BMI and depression.

To assess the potential non-linear relationship between TyG-BMI, depression, and stroke risk, Restricted Cubic Splines (RCS) were applied in the analysis. This approach allowed us to model and examine the dose–response relationship more effectively. We also conducted a validation process using two separate datasets: CHARLS as the training set and NHANES as the testing set. This approach helped assess the external validity of the model developed on the CHARLS cohort, ensuring that the findings could be generalized to an independent dataset.

In addition, a mediation effect analysis was conducted to explore the potential indirect effects of TyG-BMI and depression on stroke risk through intermediary variables, such as metabolic factors and inflammatory markers. This analysis allowed us to better understand the pathways through which TyG-BMI and depression may interact and contribute to stroke risk. Mediation analysis was performed using the appropriate statistical models, and the results were adjusted for confounding factors to ensure the validity of the findings.

Subgroup analyses were conducted based on age, gender, hypertension, diabetes, smoking, and alcohol consumption status using stratified logistic regression models. Interaction effects were assessed using likelihood ratio tests for models with and without interaction terms.

## Results

### Characteristics of the population

A total of 24,171 participants (CHARLS: 6,417; NHANES: 17,754) were included in the analysis. The clinical baseline characteristics of both groups are summarized in Table 1. Supplementary Tables S1, S2 present the clinical baseline characteristics of participants from the CHARLS and NHANES, respectively.

**Table 1:** The relationship between TyG-BMI index and depression in participants.

Characteristics	Overall	Group 1	Group 2	Group 3	Group 4	*P*
Participants, *n*	24,171	6,054	6,793	5,829	5,495	
Age (Years)	53.21 (9.84)	51.23 (10.79)	52.84 (9.12)	55.12 (9.88)	56.31 (8.07)	<0.001
Marriage, *n* (%)	18,441 (76.29)	4,918 (81.24)	5,246 (77.23)	4,252 (72.95)	4,025 (73.25)	<0.001
Gender, *n* (%)						
Female	11,656 (48.22)	3,447 (56.92)	3,291 (48.45)	2,781 (47.71)	2,137 (38.89)	<0.001
Male	12,515 (51.78)	2,607 (43.08)	3,502 (51.55)	3,048 (52.29)	3,358 (61.11)	
Educational level, *n* (%)						
Primary	16,152 (66.82)	4,203 (69.39)	4,122 (60.68)	4,171 (71.56)	3,656 (66.53)	<0.001
Secondary	5,764 (23.85)	1,220 (20.15)	1790 (26.35)	1,200 (20.59)	1,554 (28.27)	
Third	2,255 (9.33)	631 (10.42)	881 (12.97)	458 (7.86)	285 (5.19)	
Smoking, *n* (%)						
Never	15,121 (62.56)	4,028 (66.53)	4,305 (63.37)	3,407 (58.46)	3,381 (61.53)	<0.001
Ever	2,279 (9.43)	658 (10.87)	497 (8.53)	497 (8.53)	435 (7.92)	
Current	6,771 (28.01)	1,368 (22.59)	1799 (26.48)	1925 (33.02)	1,679 (30.55)	
Drinking, *n* (%)						
Never	13,826 (57.20)	3,725 (61.53)	3,995 (58.81)	3,319 (56.95)	2,787 (50.72)	<0.001
Ever	2041 (8.44)	508 (8.39)	609 (8.96)	485 (8.32)	439 (7.99)	
Current	8,304 (34.36)	1821 (30.08)	2,189 (32.22)	2025 (34.74)	2,269 (41.29)	
Hypertension, *n* (%)	93,005 (38.47)	1,570 (25.93)	3,250 (47.84)	1792 (30.74)	2,688 (48.91)	<0.001
Antihypertensive Drug, *n* (%)	7,072 (29.26)	1,168 (19.29)	2,389 (35.17)	1,400 (24.02)	2,115 (38.49)	<0.001
Diabetes, *n* (%)	3,771 (15.60)	566 (9.35)	1,279 (18.83)	619 (10.62)	1,307 (23.78)	<0.001
Antihyperglycemic Drug, *n* (%)	2,437 (10.08)	375 (6.19)	880 (12.95)	412 (7.07)	770 (14.01)	<0.001
Waist, (cm)	85.64 (11.12)	78.14 (10.02)	90.21 (11.15)	78.01 (8.61)	93.12 (12.55)	<0.001
SBP, (mmHg)	128.12 (17.32)	122.74 (18.86)	132.12 (20.36)	127.36 (18.70)	133.21 (19.31)	0.085
DBP, (mmHg)	73.90 (10.82)	71.98 (11.42)	76.24 (10.35)	72.84 (11.22)	77.43 (12.95)	0.039
Glu, (mg/dL)	111.52 (33.09)	103.23 (25.30)	118.26 (40.03)	107.31 (22.40)	119.32 (41.82)	<0.001
HDL, (mg/dL)	49.12 (17.41)	52.82 (16.02)	42.31 (29.17)	49.63 (18.35)	42.01 (17.89)	<0.001
LDL, (mg/dL)	118.72 (36.37)	116.18 (36.37)	122.39 (33.95)	116.68 (34.79)	125.25 (38.91)	<0.001
TC, (mg/dL)	197.64 (43.54)	190.34 (32.51)	201.43 (49.54)	192.27 (34.97)	203.89 (39.57)	<0.001
TG, (mg/dL)	134.08 (100.61)	89.41 (41.64)	170.18 (111.50)	97.26 (55.09)	169.18 (109.05)	<0.001
CRP, (mg/dL)	3.24 (5.21)	2.69 (5.89)	2.86 (6.40)	2.88 (7.99)	3.23 (4.11)	0.003
Stroke, *n* (%)	1,223 (5.06)	242 (4.00)	337 (4.96)	312 (5.35)	332 (6.04)	<0.001

Continuous variables are presented as mean (standard deviation). Categorical variables are presented as *n* (%). BMI, body mass index; TyG, triglyceride-glucose index; n, number; SBP, systolic blood pressure; DBP, diastolic blood pressure; SD, standard deviation; Glu, glucose; HDL, high-density lipoprotein; LDL, low-density lipoproteins; TC, total cholesterol; TG, triglyceride; CRP, C-reactive protein. Group 1: TyG-BMI < median & No Depression; Group 2: TyG-BMI ≥ median & No Depression; Group 3: TyG-BMI < median & Depression; Group 4: TyG-BMI ≥ median & Depression.

The baseline characteristic tables highlight the distribution differences across the four subgroups defined by TyG-BMI level and depression status. Participants in Group 4 (TyG-BMI ≥ median & Depression) tended to be older, predominantly male, and had the highest prevalence of diabetes (23.78%), hypertension (48.91%), and stroke (6.04%).

### The incidence rate of stroke

Table 2 shows the association between TyG-BMI, depression, and stroke. In the unadjusted model (Model 1), Group 2 (TyG-BMI ≥ median and no depression) exhibited a 64.4% increased risk of stroke compared to the normal Group 1 (TyG-BMI < median and no depression) (unadjusted OR 1.644, 95% CI 1.077–2.311, *p* < 0.001). Group 3 (TyG-BMI < median and depression) showed a 22.1% increase in stroke risk (OR 1.221, 95% CI 1.123–1.318, *p* < 0.001). Group 4 (TyG-BMI ≥ median and depression) demonstrated a 94.6% increase in stroke risk (OR 1.946, 95% CI 1.133–2.762, *p* < 0.001).

**Table 2:** The combined effect of TyG-BMI and depression on stroke risk: multivariable logistic regression analysis.

Characteristics	Model 1		Model 2		Model 3	
	OR (95%CI)	*p* value	OR (95%CI)	*p* value	OR (95%CI)	*p* value
Combined analysis of the CHARLS and NHANES cohorts						
Group 1	Ref		Ref		Ref	
Group 2	1.644 (1.077–2.311)	<0.001	1.949 (1.201–2.697)	<0.001	1.730 (1.163–2.297)	0.002
Group 3	1.221 (1.123–1.318)	<0.001	1.205 (1.109–1.301)	<0.001	1.160 (1.043–1.277)	0.027
Group 4	1.946 (1.133–2.762)	<0.001	2.068 (1.049–3.087)	<0.001	2.315 (1.503–3.126)	<0.001
CHARLS cohort						
Group 1	Ref		Ref		Ref	
Group 2	1.653 (1.126–2.179)	<0.001	1.589 (1.146–2.031)	<0.001	1.695 (1.127–2.263)	<0.001
Group 3	1.379 (1.034–1.724)	<0.001	1.358 (1.037–1.678)	<0.001	1.338 (1.094–1.582)	<0.001
Group 4	2.318 (1.379–3.256)	<0.001	2.247 (1.244–3.249)	<0.001	1.983 (1.317–2.649)	<0.001
NHANES cohort						
Group 1	Ref		Ref		Ref	
Group 2	1.505 (1.034–1.976)	<0.001	1.258 (1.012–1.503)	<0.001	1.433 (1.099–1.767)	0.002
Group 3	1.329 (1.077–1.581)	<0.001	1.174 (1.008–1.339)	<0.001	1.094 (1.007–1.181)	0.041
Group 4	1.819 (1.206–2.431)	<0.001	2.134 (1.275–2.972)	<0.001	1.830 (1.411–2.249)	<0.001

BMI, body mass index, TyG, triglyceride-glucose index; Group 1: TyG-BMI < median & No Depression; Group 2: TyG-BMI ≥ median & No Depression; Group 3: TyG-BMI < median & Depression; Group 4: TyG-BMI ≥ median & Depression. Model 1: No one was adjusted; Model 2: age, sex, marriage, education level, smoking status, drinking, hypertension and diabetes were adjusted; Model 3: Model 2 + Waist, SBP, DBP, Glu, HDL, LDL, TC, TG and CRP.

After adjusting for potential confounders (Models 2 and 3), the results remained significant. In the fully adjusted Model 3, the stroke risk for Group 2 increased by 73.0% (adjusted OR [aOR] 1.730, 95% CI 1.163–2.297, *p* = 0.002). The stroke risk for Group 3 increased by 16.0% (aOR 1.160, 95% CI 1.043–1.277, *p* = 0.002). Group 4 showed a 131.5% increase in stroke risk (aOR 2.310, 95% CI 1.503–3.126, *p* < 0.001). These results remain valid in the separate analyses of the CHARLS and NHANES cohorts (Table 2).

**Figure 2:**
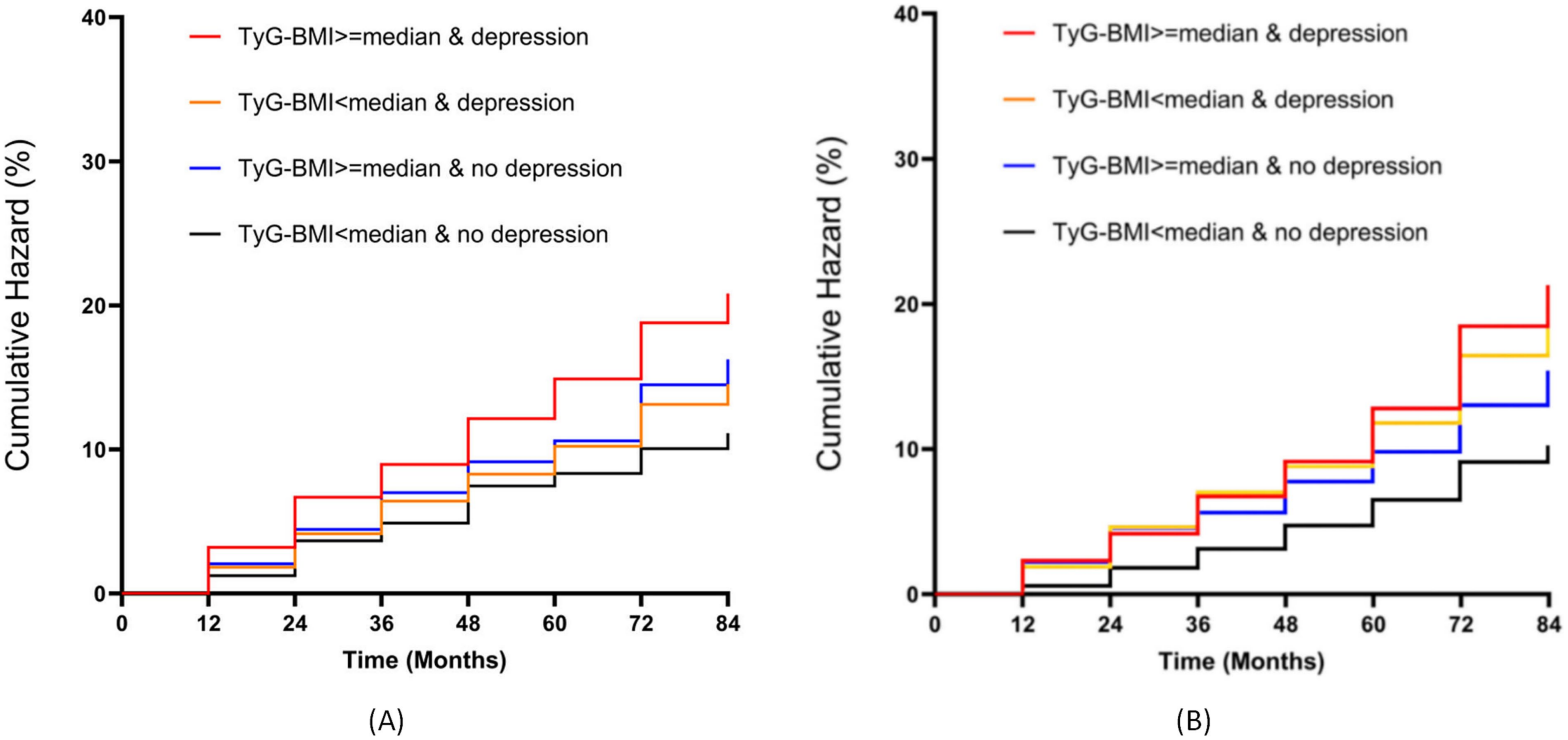
Kaplan–Meier survival analysis of cumulative stroke incidence stratified by TyG-BMI and depression status. **(A)** CHARLS cohort; **(B)** NHANES cohort.

Figure 2 displays the Kaplan–Meier curve of cumulative stroke incidence in the study population. The results from both the CHARLS and NHANES databases indicate that Group 4 (TyG-BMI ≥ median & Depression) had the highest stroke incidence.

**Figure 3:**
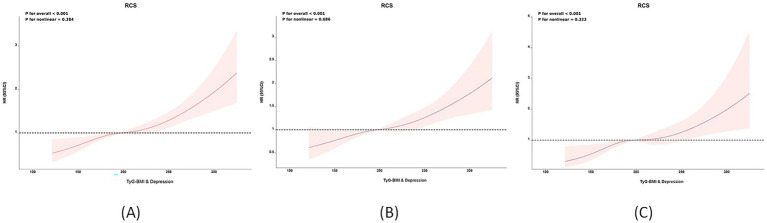
Non-linear associations between TyG-BMI combined with depression and stroke risk: Restricted cubic spline (RCS) analysis. **(A)** Overall population; **(B)** CHARLS cohort; **(C)** NHANES cohort.

When TyG-BMI was analyzed as a continuous variable, a linear positive correlation between TyG-BMI combined with depression and stroke risk was observed (RCS regression, non-linear *p*-value = 0.384; Figure 3A). Figures 3B,C also show that TyG-BMI combined with depression is positively correlated with stroke risk in both the CHARLS (Figure 3B) and NHANES (Figure 3C) in separate analyses Supplementary Table S3 highlights the heterogeneity issues within the CHARLS and NHANES cohorts through meta-regression analysis, with urban–rural residence and race showing the most significant impact on the outcomes. Supplementary Table S4 quantifies the additive interaction between TyG-BMI and depression, revealing that high TyG-BMI without depression (RERI = 1.823, AP = 0.475, S = 1.935), normal TyG-BMI with depression (RERI = 1.156, AP = 0.387, S = 1.455), and the coexposure to high TyG-BMI and depression (RERI = 2.734, AP = 0.623, S = 2.208) all demonstrate significant additive interactions. Notably, the coexposure of high TyG-BMI and depression shows a supra-additive effect, indicating that comorbid metabolic dysfunction and depression significantly elevate stroke risk beyond their individual contributions.

### Results of subgroup analysis

As shown in Table 3, in the subgroup analysis, the association between TyG-BMI, depression, and stroke risk was not influenced by gender, age, smoking status, alcohol consumption, or diabetes status in any pre-specified or exploratory subgroup. Specifically, the interaction between these variables and TyG-BMI combined with depression was not statistically significant (interaction *p*-value>0.05). However, we observed that in populations aged<60, those who had never smoked or had quit smoking, those who had never consumed alcohol, and those without diabetes, the stroke risk in Group 4 was significantly higher (*p* < 0.05). In contrast, the interaction between hypertension and TyG-BMI combined with depression was statistically significant (interaction *p*-value<0.05). Specifically, in individuals without hypertension, the risk of stroke was 2.34 times higher in those with elevated TyG-BMI and depression compared to those without both conditions. This result remained consistent in separate analyses of the CHARLS and NHANES databases (Supplementary Tables S5, S6).

**Table 3:** The effect of TyG-BMI and depression on stroke risk: subgroup analysis.

Characteristics	Group 1		Group 2		Group 3		Group 4		*P* for interaction
	OR (95%CI)	*p*	OR (95%CI)	*p*	OR (95%CI)	*p*	OR (95%CI)	*p*	
Age									0.201
<60	Ref		1.663 (1.192–2.134)	<0.001	1.886 (1.319–2.453)	<0.001	2.775 (1.816–3.734)	<0.001	
≥60			1.038 (0.813–1.264)	0.342	1.263 (0.813–1.713)	0.421	1.508 (1.124–1.891)	0.024	
Gender									0.774
Male	Ref		1.323 (0.819–1.827)	0.078	1.308 (0.876–1.739)	0.087	2.034 (1.332–2.736)	<0.001	
Female			1.479 (1.214–1.744)	0.042	1.322 (0.912–1.731)	0.239	1.944 (1.421–2.567)	<0.001	
Smoking									0.524
Never	Ref		1.632 (1.143–2.121)	<0.001	1.378 (1.132–1.624)	<0.001	1.825 (1.309–2.340)	<0.001	
Ever			1.553 (0.862–2.243)	0.432	1.892 (0.911–2.873)	0.384	2.574 (0.975–4.173)	0.375	
Current			1.251 (0.821–1.681)	0.677	1.508 (0.879–2.137)	0.471	1.531 (0.924–2.137)	0.269	
Drinking									0.417
Never	Ref		1.508 (1.187–1.828)	<0.001	1.496 (1.113–1.878)	<0.001	2.179 (1.512–2.845)	<0.001	
Ever			1.020 (0.434–1.605)	0.786	0.887 (0.455–1.318)	0.246	1.218 (0.702–1.734)	0.423	
Current			1.962 (1.298–2.625)	<0.001	1.872 (1.213–2.531)	<0.001	2.037 (1.328–2.745)	<0.001	
Hypertension									<0.001
No	Ref		1.959 (1.233–2.684)	<0.001	1.874 (1.453–2.295)	<0.001	2.349 (1.651–3.047)	<0.001	
Yes			1.048 (0.648–1.448)	0.613	1.129 (0.407–1.851)	0.346	1.344 (0.761–1.927)	0.179	
Diabetes									0.227
No	Ref		1.716 (1.298–2.134)	<0.001	1.673 (1.152–2.194)	<0.001	2.050 (1.417–2.683)	<0.001	
Yes			1.439 (0.634–2.243)	0.411	1.702 (0.872–2.531)	0.249	1.681 (0.815–2.547)	0.208	

BMI body mass index, TyG triglyceride-glucose index; Group 1: TyG-BMI < median & No Depression; Group 2: TyG-BMI ≥ median & No Depression; Group 3: TyG-BMI < median & Depression; Group 4: TyG-BMI ≥ median & Depression. Adjust for age, sex, marriage, education level, smoking status, drinking, hypertension, diabetes, Waist, SBP, DBP, Glu, HDL, LDL, TC, TG and CRP.

### Analysis of mediation effects

Table 4 presents the results of the bidirectional mediation model, analyzing the interactive mechanism between TyG-BMI and depression on stroke risk (adjusting for confounding factors such as age, gender, marital status, education level, smoking, alcohol consumption, hypertension, diabetes, Waist, SBP, DBP, Glu, HDL, LDL, TC, TG and CRP). The results showed that when depression was treated as a mediator, the total effect of TyG-BMI on stroke was −207.36 (95% CI: −310.22, −18.28; *p* < 0.001), the indirect effect was 4.97 (95% CI: −3.69, 17.51; *p* = 0.387), and the direct effect was −212.33 (95% CI: −306.53, −35.79; *p* < 0.001), with the mediation effect accounting for 3.12% (*p* = 0.431). When TyG-BMI was treated as a mediator, the total effect of depression on stroke was −51.44 (95% CI: −167.70, −2.13; *p* = 0.036), the indirect effect was 12.57 (95% CI: 2.45, 23.58; *p* < 0.001), and the direct effect was −64.01 (95% CI, −170.15, −25.71; *p* < 0.001), with the mediation effect accounting for 15.5% (*p* = 0.235).

**Table 4:** Mutual mediation effects of TyG-BMI and depression on stroke risk.

Mediation	Mediation effect (95%)				
	Total effect	Indirect effect	Direct effect	Proportion	*P* for proportion
Combined analysis of the CHARLS and NHANES cohorts					
Depression	−207.36 (−310.22, −18.28) < 0.001	4.97 (−3.69, 17.51) 0.387	−212.33 (−306.53, −35.79) < 0.001	3.12%	0.431
TyG-BMI	−51.44 (−167.70, −2.13) 0.036	12.57 (2.45,23.58) < 0.001	−64.01 (−170.15, −25.71) < 0.001	15.5%	0.235
CHARLS cohort					
Depression	−146.64 (−275.21,-42.33) < 0.001	3.45 (−2.27,10.13) 0.230	−150.09 (−272.94,-52.46) 0.002	2.23%	0.232
TyG-BMI	−40.88 (−143.70, 56.03) 0.268	9.67 (2.69,18.96) 0.002	−50.55 (−146.39,37.07) < 0.001	14.2%	0.374
NHANES cohort					
Depression	−205.93 (−324.27, −6.99) < 0.001	6.21 (−4.51, 22.14) 0.416	−212.14 (−319.76, −29.13) < 0.001	3.41%	0.465
TyG-BMI	−57.76 (−177.70, 46.02) 0.257	13.47 (1.58, 26.74) < 0.001	−71.23 (−179.28, 19.28) < 0.001	16.4%	0.196

BMI body mass index, TyG triglyceride-glucose index; Adjust for: Age, sex, marriage, education level, smoking status, drinking, hypertension, diabetes, Waist, SBP, DBP, Glu, HDL, LDL, TC, TG and CRP.

### Statistical performance of the predictive model

**Figure 4:**
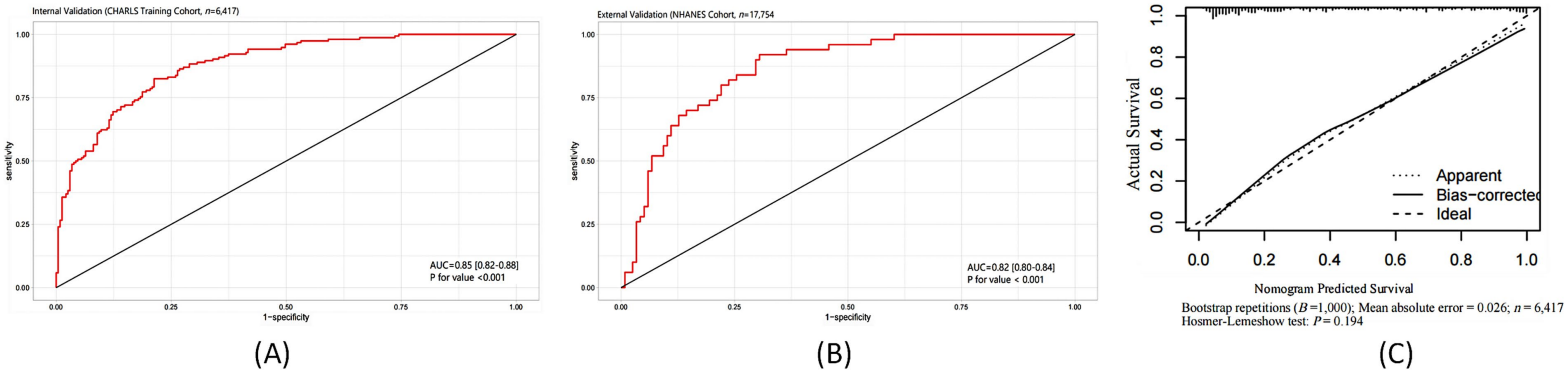
Predictive performance of the TyG-BMI combined with depression model for stroke risk: ROC curves and calibration analysis. **(A)** ROC curve in the CHARLS internal testing cohort; **(B)** ROC curve in the NHANES external validation cohort; **(C)** Calibration plot of predicted versus observed stroke probability.

To explore the predictive outcome of the TyG-BMI combined with depression on stroke occurrence, we further developed a predictive model, using the CHARLS database as the internal testing set and the NHANES database as the external validation set. This model demonstrated robust discriminatory power in both internal and external validation cohorts. As shown in Figure 4A, in the CHARLS internal testing cohort (n = 6,417), the AUC was 0.85 (95% CI: 0.82–0.88; *p* < 0.001), with sensitivity and specificity of 75% at a 1-specificity = 0.25 cutoff. As illustrated in Figure 4B, in the NHANES external validation cohort (*n* = 17,754), the model’s generalizability was confirmed, yielding an AUC of 0.82 (95% CI: 0.80–0.84; *p* < 0.001). Calibration analysis demonstrated a high concordance between the predicted probability of stroke occurrence and the observed incidence, with a mean absolute error of 0.026. The Hosmer-Lemeshow test revealed no significant deviation (*p* = 0.194), as shown in Figure 4C. The bias-corrected calibration curve closely aligned with the ideal line, further supporting the clinical reliability of the model.

## Discussion

In this study, the combination of TyG-BMI and depression was consistently associated with stroke risk across two nationally representative cohorts—CHARLS and NHANES. Our findings suggest that individuals simultaneously exposed to elevated TyG-BMI and depressive symptoms exhibited the highest risk of stroke. Moreover, TyG-BMI and depression independently contributed to further risk stratification for stroke. This association persisted even after adjusting for other well-established cardiovascular risk factors. Notably, our results indicate a potential synergistic interaction between TyG-BMI and depression in the assessment of stroke risk.

IR, as characterized by the TyG index, is recognized as a significant and modifiable risk factor for stroke (31). Such metabolic disturbances facilitate the development and progression of atherosclerosis, a fundamental pathological basis of stroke (32). Studies have shown that the association between IR and cardiovascular events, including stroke, is independent of diabetes and is particularly pronounced among individuals with obesity (33). The TyG index, initially proposed by Ramdas NV and colleagues, has been validated as a reliable, cost-effective, and straightforward surrogate marker for IR (34). It demonstrates strong correlations with traditional insulin sensitivity assessments, such as the hyperinsulinemic-euglycemic clamp technique—regarded as the gold standard for evaluating IR (35). Multiple studies have reported significant associations between TyG and IR across diverse populations. For instance, the work by Simental-Mendía et al. highlighted a strong correlation between TyG and HOMA-IR, a widely used surrogate of IR, supporting TyG as a simpler and more economical alternative (10). Similarly, in hypertensive Asian populations, TyG has been positively correlated with IR, reinforcing its relevance in Asian cohorts (36). Although some researchers have noted heterogeneity in the utility of TyG as an IR surrogate (37), its strong correlation with conventional methods makes it a practical and cost-effective tool in settings lacking advanced diagnostic capabilities.

TyG-BMI, calculated as the product of BMI and TyG, is considered a composite indicator that better reflects IR than either measure alone. It has been shown to be significantly associated with non-alcoholic fatty liver disease, cardiovascular events, prehypertension, and diabetes (34, 38, 39). A growing body of evidence also supports the association between TyG-BMI and stroke risk, potentially mediated by IR (40–42). The underlying mechanisms may involve several biological pathways: (1) Atherosclerosis—IR is implicated in the progression of atherosclerosis, endothelial dysfunction, foam cell formation, and rupture of vulnerable plaques (43); (2) Inflammatory responses—IR is frequently accompanied by a state of chronic low-grade inflammation, which accelerates atherosclerosis and promotes the release of pro-inflammatory mediators (44); and (3) Platelet dysfunction—IR alters platelet adhesion, activation, and aggregation, contributing to arterial stenosis or occlusion and consequently increasing the risk of stroke (45).

With the rapid pace of societal development and mounting life stressors, the prevalence of depression and depressive symptoms has been increasing steadily, becoming a prominent public mental health concern (46). Depression is also recognized as a critical risk factor for stroke (22). The pathophysiological mechanisms may involve dysregulation of the autonomic nervous system due to psychological stress and depressive symptoms, leading to heightened sympathetic nervous activity. Researchers have confirmed that psychological stress induced by depression activates cardiac sympathetic nerves (47), resulting in reduced coronary blood flow, increased heart rate, left ventricular hypertrophy, myocardial infarction, and sudden cardiac death (48). Furthermore, elevated cortisol levels, frequently observed in individuals with depression, are major contributors to the development of metabolic syndrome (49), which in turn is linked to pathological changes such as impaired glucose tolerance, dyslipidemia, and weight gain (50, 51). Both sympathetic overactivity and metabolic syndrome are established risk factors for stroke, thus reinforcing the association between depression and cerebrovascular events.

Subgroup analysis revealed that the combined effects of TyG-BMI and depression on stroke risk were significantly stronger in individuals without hypertension. In the non-hypertensive group, the association between TyG-BMI, depression, and increased stroke risk was significant, while in the hypertensive group, the effect was attenuated. This suggests that TyG-BMI and depression may synergistically elevate stroke risk in the absence of hypertension. The mechanism could be that, for non-hypertensive individuals, the combined metabolic (IR) and psychological (depression) stressors directly exacerbate cerebrovascular risk. Previous studies have shown that TyG-BMI and depression are independent risk factors for stroke (10, 11, 13), and their combination may contribute to stroke development by affecting vascular health and systemic inflammation (5, 52). In contrast, hypertension is a dominant risk factor, and in individuals with hypertension, it may overshadow the additional risks posed by TyG-BMI and depression. This aligns with research suggesting that hypertension can “mask” the effects of other risk factors (14). While TyG-BMI and depression significantly affect stroke risk in non-hypertensive individuals, the impact of hypertension might reduce their combined effect. These insights could guide clinicians in managing stroke risk, especially in non-hypertensive populations. Further studies are needed to explore these interactions and refine personalized prevention strategies.

This study is the first to reveal a significant positive association between the combination of TyG-BMI and depression with stroke risk, demonstrating that their joint effect surpasses that of either factor alone. The major strength of this study lies in the utilization of two large-scale, nationally representative cohorts—CHARLS and NHANES—with comprehensive and reliable clinical datasets. Moreover, this study extends prior research by confirming the positive association between elevated TyG-BMI combined with depressive symptoms and stroke risk. Unlike previous studies, our analysis examined TyG-BMI and depression both as categorical and continuous variables in relation to stroke risk, thus reducing information loss and allowing for quantitative evaluation of the association. The results robustly support our initial hypothesis.

To assess the robustness of our findings, we also conducted independent analyses of TyG-BMI and depression in relation to stroke, and found that although each was positively associated with stroke risk, their predictive value was lower when analyzed separately than when combined. These findings underscore the clinical value of jointly evaluating TyG-BMI and depression for the primary prevention of stroke.

Nevertheless, several limitations should be acknowledged. First, although based on two national databases, this study remains a cross-sectional observational analysis and therefore cannot establish a causal relationship between the combined effect of TyG-BMI and depression on stroke risk. Clinical trials are warranted to evaluate the benefits of reducing TyG-BMI or alleviating depression for the primary prevention of stroke, particularly in individuals with divergent risk profiles. Second, while PHQ-9 and CESD-10 are validated and reliable screening tools for depressive symptoms, they are not diagnostic instruments; thus, some misclassification in depression status is possible. Lastly, stroke diagnosis in this study primarily relied on self-reported data and questionnaires, rather than definitive imaging evidence, which may introduce information and recall biases. Nonetheless, previous studies and international aging surveys have validated the feasibility of using self-reported outcomes in population-based research.

## Conclusion

In conclusion, this study found a significant positive association between the combination of TyG-BMI and depression in the assessment of stroke risk. The results highlight the impact of the joint exposure to TyG-BMI and depression on stroke occurrence and suggest that, in clinical practice, the combined evaluation of TyG-BMI and depression should be used to further stratify stroke risk and optimize primary prevention strategies.

## Data Availability

The datasets presented in this study can be found in online repositories. The names of the repository/repositories and accession number(s) can be found at: One of the data sources for this study population is the China Health and Retirement Longitudinal Study (CHARLS) (http://charls.pku.edu.cn/) Another data source is the National Health and Nutrition Examination Survey (NHANES) database, a major project of the National Center for Health Statistics (NCHS) in the United States.
