# Supplementary material for: Synergistic effects of triglyceride-glucose index and body mass index combined with depression in predicting stroke events: a study based on two national cohorts
Source: Front Nutr. 2025 Sep 3;12:1633655. doi: 10.3389/fnut.2025.1633655 (PMC12442736; doi:10.3389/fnut.2025.1633655)
Supplement: Supplementary file 1 [file Table_1.docx]

Supplementary_Table S1. Characteristics of participants in the CHARLS database

|  | Overall | Group1 | Group2 | Group3 | Group4 | *P* |
| --- | --- | --- | --- | --- | --- | --- |
| Participants, n | 6417 | 1616 | 1801 | 1592 | 1408 |  |
| Age(Years) | 58.49 (9.09) | 59.7 (9.84) | 57.06 (8.54) | 59.9 (9.44) | 57.31 (8.46) | <0.001 |
| Marriage,n (%) | 5732 (89.3) | 1442 (89.2) | 1682 (93.4) | 1358 (85.3) | 1250 (88.8) | <0.001 |
| Gender, n (%) | | | | | | <0.001 |
| Female | 3085 (48.1) | 977 (60.5) | 883 (49) | 782 (49.1) | 443 (31.5) |  |
| Male | 3332 (51.9) | 639 (39.5) | 918 (51) | 810 (50.9) | 965 (68.5) |  |
| Educational level, n (%) | | | | | | <0.001 |
| Primary | 4481 (70.0) | 1127 (69.7) | 1093 (60.7) | 1234 (77.5) | 1027 (72.9) |  |
| Secondary | 1751 (27.2) | 446 (27.6) | 620 (34.4) | 333 (20.9) | 352 (25) |  |
| Third | 185 (2.8) | 43 (2.7) | 88 (4.9) | 25 (1.6) | 29 (2.1) |  |
| Smoking, n (%) | | | | | | <0.001 |
| Never | 3842 (59.9) | 813 (50.3) | 1104 (61.3) | 894 (56.2) | 1031 (73.2) |  |
| Ever | 521 (8.1) | 120 (7.4) | 186 (10.3) | 112 (7.0) | 103 (7.3) |  |
| Current | 2054 (32.0) | 683 (42.3) | 511 (28.4) | 586 (36.8) | 274 (19.5) |  |
| Drinking, n (%) | | | | | | <0.001 |
| Never | 3708 (57.8) | 867 (53.7) | 1027 (57) | 894 (56.2) | 920 (65.3) |  |
| Ever | 496 (7.7) | 110 (6.8) | 134 (7.4) | 139 (8.7) | 113 (8.0) |  |
| Current | 2213 (34.5) | 639 (39.5) | 640 (35.5) | 559 (35.1) | 375 (26.6) |  |
| Hypertension, n (%) | 2395 (37.3) | 451 (27.9) | 837 (46.5) | 451 (28.3) | 656 (46.6) | <0.001 |
| Antihypertensive Drug，n (%) | 939 (14.6) | 98 (6.1) | 374 (20.8) | 143 (9.0) | 324 (23) | <0.001 |
| Diabetes, n (%) | 889 (13.9) | 104 (6.4) | 341 (18.9) | 139 (8.7) | 305 (21.7) | <0.001 |
| Antihyperglycemic Drug，n (%) | 190 (3.0) | 16 (1.0) | 67 (3.7) | 22 (1.4) | 85 (6.0) | <0.001 |
| Waist, (cm) | 83.77 (12.32) | 77.76 (9.80) | 89.83 (12.11) | 77.51 (9.48) | 89.88 (11.02) | <0.001 |
| SBP, (mmHg) | 129.60 (21.07) | 126.64 (20.39) | 133.51 (20.80) | 125.48 (20.65) | 132.65 (21.30) | 0.085 |
| DBP, (mmHg) | 75.44 (12.09) | 73.17 (11.50) | 78.21 (11.84) | 72.47 (11.66) | 77.88 (12.25) | 0.039 |
| Glu, (mol/dL) | 109.56 (35.05) | 102.17 (23.87) | 116.81 (40.67) | 101.86 (23.93) | 117.45 (43.88) | <0.001 |
| HDL, (mg/dL) | 51.81 (15.36) | 57.43 (14.72) | 45.51 (12.45) | 58.04 (16.18) | 46.34 (13.15) | <0.001 |
| LDL, (mg/dL) | 116.63 (35.12) | 113.68 (32.47) | 118.60 (36.61) | 113.72 (32.15) | 120.78 (38.60) | <0.001 |
| TC, (mg/dL) | 193.75 (38.34) | 186.97 (36.57) | 198.32 (37.50) | 188.75 (36.52) | 201.36 (41.16) | <0.001 |
| TG , (mg/dL) | 129.39 (94.46) | 87.91 (39.90) | 168.07 (109.08) | 93.82 (46.71) | 167.73 (119.93) | <0.001 |
| CRP, (mg/dL) | 2.58 (6.89) | 2.42 (6.47) | 2.66 (6.79) | 2.73 (8.67) | 2.47 (4.94) | 0.003 |
| Stroke, n(%) | 282 (4.4) | 49 (3.0) | 95 (5.3) | 61 (3.8) | 77 (5.5) | <0.001 |

Continuous variables are presented as mean (standard deviation). Categorical variables are presented as n (%).

BMI body mass index, TyG triglyceride-glucose index, n number, SBP systolic blood pressure, DBP diastolic blood pressure, SD standard deviation, Glu glucose, HDL high-density lipoprotein, LDL low-density lipoproteins, TC total cholesterol,

TG triglyceride, CRP C-reactive protein

Group1: TyG-BMI < median & No Depression; Group2: TyG-BMI ≥ median & No Depression;

Group3: TyG-BMI < median & Depression; Group4: TyG-BMI ≥ median & Depression

Supplementary_Table S2. Characteristics of participants in the NHANES database

|  | Overall | Group1 | Group2 | Group3 | Group4 | *P* |
| --- | --- | --- | --- | --- | --- | --- |
| Participants, ,n (%) | 17754 | 4438 (24.99) | 4992 (28.12) | 4237 (23.87) | 4087 (23.02) |  |
| Age (SD) | 51.32 (8.49) | 47.21 (11.84) | 50.33 (9.63) | 52.3 (10.44) | 55.29 (7.67) | <0.001 |
| Marriage,n (%) | 12709 (71.58) | 3476 (78.23) | 3564 (71.42) | 2894 (68.31) | 2775 (67.89) | <0.001 |
| Gender, n (%) | | | | | | <0.001 |
| Female,n (%) | 8571 (48.28) | 2470 (55.65) | 2408 (48.23) | 1999 (47.19) | 1694 (41.46) |  |
| Male,n (%) | 9183 (51.72) | 1968 (44.35) | 2584 (51.77) | 2238 (52.81) | 2393 (58.54) |  |
| Educational level, n (%) | | | | | | <0.001 |
| Primary,n (%) | 11671 (65.74) | 3076 (69.31) | 3029 (60.68) | 2937 (69.32) | 2629 (64.33) |  |
| Secondary,n (%) | 4013 (22.60) | 774 (17.43) | 1170 (23.44) | 867 (20.46) | 1202 (29.41) |  |
| Third,n (%) | 2070 (11.66) | 588 (13.26) | 793 (15.88) | 433 (10.22) | 256 (6.26) |  |
| Smoking, n (%) | | | | | | <0.001 |
| Never,n (%) | 11279 (63.53) | 3215 (72.43) | 3201 (64.13) | 2513 (59.33) | 2350 (57.51) |  |
| Ever,n (%) | 1758 (9.90) | 538 (12.13) | 503 (10.07) | 385 (9.09) | 332 (8.13) |  |
| Current,n (%) | 4717 (23.57) | 685 (15.44) | 1288 (25.80) | 1339 (31.58) | 1405 (34.36) |  |
| Drinking, n (%) | | | | | | <0.001 |
| Never,n (%) | 10118 (56.99) | 2858 (64.37) | 2968 (59.46) | 2425 (57.23) | 1867 (45.69) |  |
| Ever,n (%) | 1545 (8.71) | 398 (8.98) | 475 (9.52) | 346 (8.17) | 326 (7.97) |  |
| Current,n (%) | 6091 (34.31) | 1182 (26.65) | 1549 (31.02) | 1466 (34.6) | 1894 (46.34) |  |
| Hypertension, n (%) | 6905 (38.89) | 1119 (24.96) | 2413 (48.34) | 1341 (31.65) | 2032 (49.73) | <0.001 |
| Antihypertensive Drug，n (%) | 6133 (35.54) | 1070 (24.11) | 2015 (40.38) | 1257 (29.67) | 1791 (43.81) | <0.001 |
| Diabetes, n (%) | 2882 (16.23) | 462 (10.4) | 938 (18.79) | 480 (11.34) | 1002 (24.52) | <0.001 |
| Antihyperglycemic Drug，n (%) | 2247 (12.66) | 359 (8.10) | 813 (16.28) | 390 (9.21) | 685 (16.77) | <0.001 |
| Waist, (cm, SD) | 86.41 (10.21) | 79.31 (10.12) | 92.32 (10.19) | 79.49 (7.73) | 94.68 (13.02) | <0.001 |
| SBP, (mmHg, SD) | 127.49 (14.31) | 121.34 (17.33) | 130.41 (19.92) | 129.24 (16.75) | 134.76 (17.32) | 0.079 |
| DBP, (mmHg, SD) | 73.32 (10.19) | 71.12 (9.45) | 74.26 (8.87) | 73.21 (10.78) | 76.98 (13.65) | 0.064 |
| Glu, (mol/dL, SD) | 112.45 (32.13) | 104.29 (26.73) | 119.72 (39.39) | 112.76 (20.87) | 121.19 (39.76) | <0.001 |
| HDL, (mg/dL, SD) | 48.31 (19.47) | 51.12 (16.71) | 41.46 (37.89) | 47.21 (19.21) | 40.32 (19.27) | <0.001 |
| LDL, (mg/dL, SD) | 119.32 (37.62) | 118.77 (40.27) | 126.17 (31.28) | 119.63 (37.42) | 129.71 (39.21) | <0.001 |
| TC, (mg/dL, SD) | 199.45 (48.74) | 192.71 (29.47) | 203.54 (61.57) | 195.79 (33.42) | 206.41 (37.98) | <0.001 |
| TG , (mg/dL, SD) | 136.77 (104.76) | 93.91 (43.37) | 177.28 (113.91) | 102.71 (63.47) | 172.64 (98.17) | <0.001 |
| CRP, (mg/dL, SD) | 3.49 (4.37) | 2.97 (5.31) | 3.05 (6.02) | 3.02 (7.33) | 3.98 (3.27) | 0.003 |
| Stroke, n(%) | 941 (5.3) | 193 (4.35) | 242 (4.85) | 251 (5.92) | 255 (6.24) | <0.001 |

Continuous variables are presented as mean (standard deviation). Categorical variables are presented as n (%).

BMI body mass index, TyG triglyceride-glucose index, n number, SBP systolic blood pressure, DBP diastolic blood pressure, SD standard deviation, Glu glucose, HDL high-density lipoprotein, LDL low-density lipoproteins, TC total cholesterol,

TG triglyceride, CRP C-reactive protein

Group1: TyG-BMI < median & No Depression; Group2: TyG-BMI ≥ median & No Depression;

Group3: TyG-BMI < median & Depression; Group4: TyG-BMI ≥ median & Depression

Supplementary_Table S3. Heterogeneity Analysis of the CHARLS and NHANES Cohorts (Meta-regression Results)

| Characteristics | β (SE)​ | ​z | ​*P* | ​I² (%)​ |
| --- | --- | --- | --- | --- |
| Age | 0.32 (0.12) | 2.67 | 0.008 | 38.2 |
| Gender | -0.15 (0.08) | -1.88 | 0.060 | 12.7 |
| Educational level | 0.24 (0.11) | 2.41 | 0.002 | 24.6 |
| Residence | 0.41 (0.15) | 2.73 | 0.006 | 67.5 |
| Race | 0.37 (0.14) | 2.64 | 0.008 | 72.3 |

Supplementary_Table S4. Additive Interaction Indices of TyG-BMI and Depression

| Exposure combinations | RERI (95%CI) | AP (95%CI) | S (95%CI) |
| --- | --- | --- | --- |
| High TyG-BMI (without depression) | 1.823 (1.433, 2.213) | 0.475 (0.313, 0.637) | 1.935 (1.547, 2.324) |
| Depression (with normal TyG-BMI) | 1.156 (0.827, 1.486) | 0.387 (0.225, 0.548) | 1.455 (1.159, 1.751) |
| High TyG-BMI & depression | 2.734 (2.154, 3.313) | 0.623 (0.512, 0.734) | 2.208 (1.892, 2.523) |

Supplementary_Table S5. Subgroup analysis of the effect of the TyG-BMI index and Depression on the risk of Stroke based on the CHARLS database.

|  | Group1 | | Group2 | | Group3 | | Group4 | | *P* for interaction |
| --- | --- | --- | --- | --- | --- | --- | --- | --- | --- |
|  | HR(95%CI) | *P* | HR(95%CI) | *P* | HR(95%CI) | *P* | HR(95%CI) | *P* |  |
| Age |  | | | | | | | | 0.176 |
| <60 | Ref | | 1.614 (1.304-1.923) | <0.001 | 1.841 (1.440-2.242) | <0.001 | 2.726 (1.928-3.523) | <0.001 |  |
| ≥60 |  |  | 1.088 (0.701-1.475) | 0.231 | 1.214 (0.925-1.502) | 0314 | 1.553 (1.335-1.770) | <0.001 |  |
| Gender |  | | | | | | | | 0.643 |
| Male | Ref | | 1.373 (0.707-2.038) | 0.131 | 1.258 (0.665-1.850) | 0.213 | 2.079 (1.543-2.615) | <0.001 |  |
| Female |  |  | 1.529 (1.425-1.633) | <0.001 | 1.327 (0.801-1.852) | 0.134 | 2.044 (1.632-2.455) | <0.001 |  |
| Smoking |  | | | | | | | | 0.312 |
| Never | Ref | | 1.637 1.264-2.009) | <0.001 | 1.423 (1.343-1.503) | <0.001 | 1.870 (1.520-2.219) | <0.001 |  |
| Ever |  |  | 1.548 (0.741-2.354) | 0.213 | 1.888 (0.790-2.985) | 0.475 | 2.569 (0.854-4.284) | 0.264 |  |
| Current |  |  | 1.256 (0.710-1.802) | 0.432 | 1.513 (0.767-2.258) | 0.362 | 1.581 (0.813-2.348) | 0.154 |  |
| Drinking |  | | | | | | | | 0.326 |
| Never | Ref | | 1.513 (1.308-1.717) | <0.001 | 1.541 (1.324-1.757) | <0.001 | 2.224 (1.723-2.724) | <0.001 |  |
| Ever |  |  | 1.019 (0.322-1.716) | 0.671 | 0.886 (0.343-1.429) | 0.132 | 1.213 (0.581-1.845) | 0.212 |  |
| Current |  |  | 2.011 (1.509-2.513) | <0.001 | 1.922 (1.424-2.419) | 0.004 | 1.992 (1.449-2.534) | <0.001 |  |
| Hypertension |  | | | | | | | | <0.001 |
| NO | Ref | | 2.008 (1.444-2.572) | <0.001 | 1.879 (1.574-2.184) | <0.001 | 2.394 (1.862-2.926) | <0.001 |  |
| YES |  |  | 1.053 (0.537-1.569) | 0.327 | 1.129 (0.295-1.962) | 0.435 | 1.339 (0.640-2.038) | 0.264 |  |
| Diabetes |  | | | | | | | | 0.134 |
| NO | Ref | | 1.671 (1.419-1.923) | <0.001 | 1.628 (1.273-1.983) | <0.001 | 2.100 (1.628-2.571) | <0.001 |  |
| YES |  |  | 1.488 (0.845-2.131) | 0.345 | 1.706 (0.993-2.419) | 0.357 | 1.726 (0.694-2.758) | 0.176 |  |

BMI body mass index, TyG triglyceride-glucose index;

Group1: TyG-BMI < median & No Depression; Group2: TyG-BMI ≥ median & No Depression;

Group3: TyG-BMI < median & Depression; Group4: TyG-BMI ≥ median & Depression

Adjust for age, sex, marriage, education level, smoking status, drinking, hypertension, diabetes, Waist, SBP, DBP, Glu, HDL, LDL, TC, TG and CRP

Supplementary_Table S6. Subgroup analysis of the effect of the TyG-BMI index and Depression on the risk of Stroke based on the NHANES database.

|  | Group1 | | Group2 | | Group3 | | Group4 | | *P* for interaction |
| --- | --- | --- | --- | --- | --- | --- | --- | --- | --- |
|  | HR(95%CI) | *P* | HR(95%CI) | *P* | HR(95%CI) | *P* | HR(95%CI) | *P* |  |
| Age |  | | | | | | | | 0.092 |
| <60 | Ref | | 1.566 (1.282-1.850) | <0.001 | 1.910 (1.348-2.471) | <0.001 | 3.205 (1.615-4.794) | <0.001 |  |
| ≥60 |  |  | 1.678 (0.821-2.535) | 0.164 | 1.210 (0.783-1.636) | 0.382 | 1.356 (1.218-1.494) | <0.001 |  |
| Gender |  | | | | | | | | 0.431 |
| Male | Ref | | 1.459 (0.865-2.052) | 0.178 | 1.204 (0.763-1.644) | 0.214 | 1.928 (1.124-2.732) | <0.001 |  |
| Female |  |  | 1.277 (1.201-1.353) | <0.001 | 1.490 (1.282-1.697) | <0.001 | 1.922 (1.170-2.674) | 0.004 |  |
| Smoking |  | | | | | | | | 0.437 |
| Never | Ref | | 1.526 (1.443-1.608) | <0.001 | 1.404 (1.257-1.550) | <0.001 | 1.675 (1.022-2.328) | <0.001 |  |
| Ever |  |  | 1.284 (0.781-1.787) | 0.127 | 1.611 (0.643-2.578) | 0.217 | 2.416 (0.677-4.155) | 0.312 |  |
| Current |  |  | 1.065 (0.496-1.633) | 0.426 | 1.236 (0.784-1.688) | 0.231 | 1.360 (0.638-2.082) | 0.384 |  |
| Drinking |  | | | | | | | | 0.431 |
| Never | Ref | | 1.562 (1.344-1.780) | <0.001 | 1.498 (1.185-1.810) | <0.001 | 2.427 (1.606-3.247) | <0.001 |  |
| Ever |  |  | 0.864 (0.437-1.291) | 0.554 | 1.060 (0.552-1.567) | 0.643 | 1.182 (0.883-1.481) | 0.347 |  |
| Current |  |  | 1.904 (1.335-2.472) | <0.001 | 1.742 (1.160-2.323) | <0.001 | 2.022 (1.460-2.583) | <0.001 |  |
| Hypertension |  | | | | | | | | 0.007 |
| NO | Ref | | 1.978 (1.175-2.780) | <0.001 | 1.669 (1.402-1.935) | <0.001 | 2.322 (1.683-2.961) | <0.001 |  |
| YES |  |  | 0.962 (0.648-1.276) | 0.532 | 1.141 (0.680-1.602) | 0.147 | 1.417 (0.738-2.095) | 0.433 |  |
| Diabetes |  | | | | | | | | 0.314 |
| NO | Ref | | 1.835 (1.475-2.195) | <0.001 | 1.673 (1.343-2.002) | <0.001 | 2.239 (1.128-3.349) | <0.001 |  |
| YES |  |  | 1.681 (0.326-3.036) | 0.326 | 2.195 (0.765-3.625) | 0.241 | 1.539 (0.653-2.424) | 0.274 |  |

BMI body mass index, TyG triglyceride-glucose index;

Group1: TyG-BMI < median & No Depression; Group2: TyG-BMI ≥ median & No Depression;

Group3: TyG-BMI < median & Depression; Group4: TyG-BMI ≥ median & Depression

Adjust for age, sex, marriage, education level, smoking status, drinking, hypertension, diabetes, Waist, SBP, DBP, Glu, HDL, LDL, TC, TG and CRP
